# Gaps in Measuring and Mitigating Implicit Bias in Healthcare

**DOI:** 10.3389/fphar.2021.633565

**Published:** 2021-03-17

**Authors:** Sally A. Arif, Jessica Schlotfeldt

**Affiliations:** ^1^ Department of Pharmacy Practice, Midwestern University, Downers Grove, IL, United States; ^2^ Department of Pharmacy, Rush University Medical Center, Chicago, IL, United States

**Keywords:** implicit bias, diversity and inclusion, mitigating strategies, health equity, patient care

## Introduction

No one is immune to having implicit biases, including healthcare professionals. The evidence indicates that healthcare professionals exhibit the same levels of implicit bias as the wider population (FitzGerald, 2017). These unintentional biases can harm provider-patient interactions and further contribute to health inequities (Chapman et al., 2013). Organizations and accrediting bodies, including the Institute of Medicine (IOM) and The Joint Commission, recognize the importance of identifying implicit bias in order to provide a higher quality and more equitable healthcare environment for all patients (The Joint Commission, 2020).

Implicit biases are formed based on messaging and associations that become stored in our unconscious. Unconscious bias can arise when our amydala is activated and results in a fast automatic evaluation and response to socially relevant stimuli our brain receives. The result of the amygala’s processing leads to us making categorizing certain people around us that don’t necessary align with our consciously held values or beliefs. Implicit biases can alter our perception and therefore affect our ability to actively listen, have a non-judgmental attitude, make objective decisions, and communicate effectively with others. Bias doesn’t come from a place of direct intent to do or cause harm to others and experts in a field are more likely to have a “bias blind spot” as they might struggle more to identify their own biases. Dror, et al. describe eight sources of bias that can arise (Dror, 2020). While there are many types of bias, Dror and colleagues describe eight different components of bias that fall into three categories: case specific bias (category A), environmental, cultural, and experiential bias (category B), and natural bias (category C). An example of experiential bias in healthcare is when Black patients are assumed to be drug seeking because of chronic use of pain medications or frequent emergency department visits for pain crisis in the setting of sickle cell disease.

Cultural humility, an ongoing process of self-reflection, allows us to combat sources of bias, such as racism, in healthcare. By practicing a deep and intentional look inward we can start to uncover our own biases and prejudices. As healthcare professionals we need to develop meaningful and lasting strategies to mitigate our bias in order to provide equitable care to our patients. When interacting with patients we can actively practice unbiasing strategies to gain awareness of our biases and take action to redirect stereotypical responses and automatic assumptions. While there is a clear need to address the role implicit biases play in healthcare, the research demonstrates there is a lack in homogeneity when it comes to measuring implicit bias in healthcare and strategies utilized to reduce bias.

### Measuring Implicit Bias in Healthcare Practice

Recent studies have shown that healthcare providers hold unconscious bias through the use of the Implicit Association Test (IAT). The IAT was developed in 1998 and is the now the most readily available computerized online tool used to measure and bring awareness to unconscious bias in published litertature (Greenwald et al., 1998). The IAT measures the response time of unconscious associations based on various traits including, but not limited to: race, disability, gender, and ethnicity (Greenwald et al., 1998). The IAT requires the participant to categorize stimuli, such as words, labels, and pictures, into opposing classifications as quickly as possible. For example, a participant could express a faster reaction between a negative word, like “violence” and a picture of a Black face vs. a White face.

Utilizing the IAT, a multitude of research has examined clinician’s implicit bias and created discussion around how this can impact patient care. A study by Green, et al. showed clinicians associate black patients with being less cooperative with treatment compared to white patients and that black patients experience less patient centered care from clinicians with greater implicit bias (Green et al., 2007; Cooper et al., 2012; Blair et al., 2013; Oliver et al., 2014). Another study by Penner, et al. aimed to examine the relationship of oncologists who did not self-identify as black with patients who had a confirmed diagnosis of breast, colorectal, or lung cancer and self-identified as black or African American (Penner et al., 2016). Prior to seeing the patient, providers were administered the IAT and their implicit racial bias was scored. Results showed that patients who were seen by providers with higher implicit bias scores felt their appointment was not patient centered and felt it was more difficult to remember what they had discussed during the appointment. Further when the appointment was reviewed, providers with greater implicit bias practiced less supportive communication skills (*p* < 0.01) and spent a significantly shorter amount of time interacting with patients (*p* = 0.02) (Penner et al., 2016).

Although the IAT is widely utilized in research its validity and applicability has been questioned. Many psychologists have criticized the IAT for its arbitrary scoring system, its provision of results that are left to be interpreted differently by each individual, and the reality that an individual’s score can change based off of their surroundings (Azar, 2008). Reviews of research utilizing the IAT show that there is a lack of evidence showing that the test is a valid predictor of behaviors and explicit outcomes (Blanton et al., 2009; Schimmack, 2019). Regardless of critiques, the IAT is still a popular, widely used tool in identification of implicit bias, and it may prove to be useful when incorporated into a meaningful diversity and inclusion curriculum (Marcelin et al., 2019). While it can be utilized to measure unintentional preferences that can impact patient care decisions, we need more scholarship of discovery around the degree IAT predicts behavior and the changes of results over a longitudinal period of time.

### Debiasing Strategies Used in Healthcare

As awareness of clinician implicit bias and its impact on patient care and healthcare disparities grows, a push to provide education and training against implicit bias are also being employed throughout the US. Recently the state of California has enacted legislation requiring healthcare providers to participate in implicit bias training, as many institutions across the US already require (Hagiwara et al., 2020). Current research aims to address the issue of how to effectively discuss and unlearn personal implicit bias. Using tools such as the IAT and its variations can be useful in helping clinicians recognize their unconscious, implicit bias, but is recognizing bias enough to make a difference in behaviors? Current research shows that use of the IAT in conjunction with facilitated discussions on bias may be a more useful technique in dispelling bias than using the IAT alone (Stone and Moskowitz, 2011; Hagiwara et al., 2020). One study examined how the use of IATs followed by facilitated discussion impacted emergency medicine residents’ views on their own implicit bias (Zeidan et al., 2018). The residents filled out an online survey which focused on their awareness of personal bias and the topic of implicit bias pre and post IAT. The outcome post IAT and discussion showed residents were significantly more aware of their individual implicit bias (*p* = 0.003) and how this can influence delivery of care (*p* = 0.03). However, there were participants who were skeptical of the results and expressed that they were upset about the lack of neutrality in the results (Zeidan et al., 2018). Other studies recommend using “mindfulness practice” to help eliminate many of the cognitive stressors associated with implicit bias and the potential to reduce perpetration of one’s bias onto others. Implicit bias scores from the IAT were lower in participants who participated in mindful meditation sessions (Burgess et al., 2017). Data relevant to the concept of implementing the IAT in conjunction with supplemental learning techniques for successful anti-bias training is growing, however there is still a lack of uniform anti-bias trainings implemented among healthcare professionals.

Recognizing one’s own bias is difficult to do and is only one component of bias mitigation. Research shows that even if a conscious effort is made to uncover our own biases, it cannot fully mitigate implicit biases. Self-awareness does require one to start recognizing a flaw in thinking and admit to tendencies that maybe consciously unrealized. This can be especially difficult for healthcare providers as oaths are taken to “do no harm” whether it be unintentional or not. However, this is what is required in order to start making change both within ourselves and within a system that has allowed implicit bias to impact patient care and further health inequity gaps.

## Discussion

The provision of bias free healthcare should become a habit, developed through a continuous process of reflection, training, and feedback. The COVID-19 pandemic combined with the social justice movements being seen in 2020 have created a space for self-reflection in which individuals, policymakers, and leaders in healthcare must answer difficult questions about structural racism in healthcare and health inequities. This reflection requires everyone to look at how they may be contributing to systemic racism and commit to meaningful action to reduce health disparities. It is crucial that each and every healthcare professional take responsibility and learn to meaningfully address their bias in order to provide the highest quality of care to all patients. While awareness and training are first steps to reducing the potential for bias in clinical practice, other debiasing techniques can be used longitudinally such as, considering all possible scenarios, hypothesis and outcomes (Dror et al., 2015).

While the IAT has been used readily in several studies to measure the impact of implicit bias in healthcare, it comes with limitations. While studies that use IAT to measure implicit bias related to one facet of a person’s identity, it is important to recognize that intersectionality of identities within one individual, such as gender, age, sexual orientation, national origin, and disability status, can change how bias is recognized by tools like the IAT. Published literature continues to expand related to addressing stereotyping and bias in health practices, but there is still room to improve approaches when working with vulnerable or marginalized populations who are at highest risk for health disparities (Chapman et al., 2013; FitzGerald and Hurst, 2017).

There is no “one and done” approach to training when it comes to reducing implicit biases, but rather a mindful commitment and desire for progress. It is important that unconscious bias is addressed in various settings beginning at the educational level and continuing through the entirety of one’s career and lifetime. Healthcare inequality gaps continue to broaden and the issues surrounding racial bias are prevalent. The need for bias training is evident and should be incorporated into the systems that have allowed unconscious thinking and behavior to negatively impact patient care. Opportunities to translate research into policy and practice are needed in order to move the needle toward a more equitable healthcare delivery.
